# Is healthcare a ‘Necessity’ or ‘Luxury’? an empirical evidence from public and private sector analyses of South-East Asian countries?

**DOI:** 10.1186/s13561-014-0038-y

**Published:** 2015-02-11

**Authors:** Jahangir AM Khan, Rashidul Alam Mahumud

**Affiliations:** 1 Health Economics and Financing Research Group, Centre of Equity and Health Systems, icddr,b, 68 Shahid Tajuddin Ahmed Sharani, Mohakhali, Dhaka – 1212 Bangladesh; 2 Centre for Excellence in Universal Health Coverage at icddr,b and James P Grant School of Public Health, BRAC University, Dhaka, Bangladesh; 3 Adjunct Researcher, Health Economics Unit, Department of Learning, Informatics, Management and Ethics (LIME), Karolinska Institutet, SE-171 77 Stockholm, Sweden

**Keywords:** Income-elasticity, Healthcare expenditure, Public- and private sectors, Fixed- and Random effect models, South-East Asian Region, Universal health coverage

## Abstract

South-East Asian Regional (SEAR) countries range from low- to middle-income countries and have considerable differences in mix of public and private sector expenditure on health. This study intends to estimate the income-elasticities of healthcare expenditure in public and private sectors separately for investigating whether healthcare is a ‘necessity’ or ‘luxury’ for citizens of these countries. Panel data from 9 SEAR countries over 16 years (1995-2010) were employed. Fixed- and random-effect models were fitted to estimate income-elasticity of public, private and total healthcare expenditure. Results showed that one percent point increase in GDP per capita increased private expenditure on healthcare by 1.128%, while public expenditure increased by only 0.412%. Inclusion of three-year lagged variables of GDP per capita in the models did not have remarkable influence on the findings. The citizens of SEAR countries consider healthcare as a necessity while provided through public sector and a luxury when delivered by private sector. By increasing the public provisions of healthcare, more redistribution of healthcare resources can be ensured, which can accelerate the journey of SEAR countries towards universal health coverage.

## Background

In the World Health Assembly, the World Health Organization (WHO) urges (among other points) the member states “to ensure that health-financing systems include a method for prepayment of financial contributions for health care, with a view to sharing risk among the population and avoiding catastrophic health-care expenditure and impoverishment of individuals as a result of seeking care” [1]. In the low- and middle-income countries (LMICs), in absence of social health insurance, government budget is the largest share for funding healthcare where pre-payment financial contribution with scope of risk pooling mechanism is incorporated. The role of government on financing healthcare is thus a major concern in LMICs, including most of the countries in South-East Asian Region (SEAR), for achieving universal health coverage.

Different mix of public and private expenses constitutes the total health expenditure (THE) in any country [2]. The commonly found healthcare financing agents are General Government Expenditure on Health (GGEH), Ministry of Health (MoH) and households (out-of-pocket expenditure). In addition, social security fund, private health insurance and NGOs are financing healthcare in some of the countries. GGEH is the largest funder in almost all countries in SEAR, followed by Ministry of Health. Households spend a major share of THE in Bangladesh, India, Maldives and Indonesia. The trends of THE in these countries are influenced by the private expenditure on health [2].

The governments of LMICs generally allocate small share of their budget on health, while people invest more on health privately, especially as out-of-pocket payments [2]. It needs to notice here that in response to the Structural Adjustment Programmes^a^ of the World Bank and the International Monetary Fund, many developing countries since 1980s reduced public spending on health and promoted privatization and foreign competition, which can be a strong reason of increase in private expenditure on health in LMICs, including in a number of SEAR countries.

People in different income levels within a country may demand healthcare differently and they may also respond differently to their income changes [3]. Further, the effects of income changes are likely to be different on healthcare expenditure in private and public sectors since co-payments (i.e. out-of-pocket) are generally higher for healthcare from private providers. Costa-Font et al. (2009) argued that “if healthcare is a necessity that necessitates more redistribution of healthcare resources and arguably greater public involvement in healthcare” [4]. It was further argued that “the value of income elasticity provides insight into the optimal level of health expenditures in the economy and the efficient proportion of public and private health spending” [4]. Considering the variations in income level and different mix of private and public sector involvement in healthcare in SEAR countries, this study intends to estimate the income-elasticities of healthcare expenditure in public and private sectors separately for investigating whether healthcare is a ‘luxury’ or ‘necessity’ for citizens of these countries^b^.

### Previous studies

This section provides an overview of what kinds of studies on income-elasticity of healthcare were carried out and the general findings from those studies. Several earlier studies estimated the income-elasticity of healthcare using country-level data, which showed strong and positive relationship between national income and aggregate expenditure on health [5-17]. Most of those studies investigated the developed countries and the general finding was that income-elasticity estimates exceeded unity, implying that healthcare was a luxury good [5-17]. However, Farag et. al. (2012) studied the low-, middle- and high-income countries separately and found that the low-income countries were least responsive to income-levels and the middle-income countries were the most responsive ones [16]. All of these three categories of countries had lower income-elasticity than unity, implying that healthcare was a necessity. In estimating the income-elasticity of healthcare, a number of control variables, like demographic structure, health conditions etc. were considered in the regression models [5-17]. The most well-known study on income-elasticity of healthcare was done by Newhouse (1977), which included data only for one year from 13 developed countries [6]. This study found that healthcare was a ‘luxury’ (elasticity more than one). In a more recent commentary, Newhouse (2006) supported the view that organizational factors of healthcare delivery and financing mechanisms had a significant role on the magnitude of healthcare expenditure [18]. Gerdtham (1992), using cross-sectional data from OECD countries in 1985, estimated the income-elasticity to more than unity [17]. Getzen (2000) attempted to resolve the debate on relationship between income and health expenditure by estimating income-elasticity using nested multilevel model and found that healthcare was a luxury at country-level and necessity at individual-level [19]. While most of the studies in this area included developed countries in their analysis, few took even the developing countries into consideration [16,20]. By analyzing data from 173 countries for period 1995-2006, Fagar et al. (2012) observed that healthcare was a necessity in the low- and high-income countries with income-elasticity of 0.515 and 0.644 respectively [16]. Xu and Saksena (2011) analyzed data from 143 countries, separated into income groups over period 1995-2008 and found healthcare in low-income countries as a luxury and in middle- and high-income countries as a necessity [20]. Many of the studies mentioned above generally controlled for a number of variables, such as, sex- and age-structure, health systems, unobserved heterogeneity when estimated the income-elasticity of healthcare [5,16,19,20].

In sum, previous studies, which investigated the determinants of health expenditure and estimated the income-elasticity of healthcare, mostly studied the health expenditure in total, not public and private spending separately. Inference about the relationship between income and health expenditure in public and public sectors was therefore difficult to make from those studies directly. It is thus important to disaggregate THE into public and private sectors for understanding, if and to what extent health expenditure in private and public sectors are sensitive to income changes. As observed in the above-mentioned literature, most of the studies found that healthcare was a luxury though some found an opposite relationship between income and health expenditure. However, the income-elasticity of private and public expenditure on healthcare had not been studied enough though the decision making procedure on health spending in those two sectors are quite different and the impact of income change on such spending are also likely to be different.

### Conceptual framework

Change in income influences the consumption behavior of households as well as resource allocation decision of any nations. Healthcare is generally provided by both public and private sectors with different degrees of mix in different countries. Households with higher income level have more freedom of choice while making decision on healthcare purchase. It is thus more likely that households with higher income choose healthcare from private sector where quality (shorter waiting time, clean facilities etc.) of care might be better though price is high. It is expected that higher income may results in significantly higher healthcare expenditure. On the contrary, people with low-income are more likely to choose public healthcare services due to lower out-of-pocket payments (user fee). Any increase in income of low-income people may thus increase healthcare expenditure, but at a lower rate. Further, the government’s resource allocation in healthcare is subject to budget negotiation with other ministries (like, education, defense, housing etc.), which generally takes a longer time. Consequently, government may not put a proportionally high priority in health as response to the national income increase. It is thus expected that private expenditure on health which is mostly decided by households may increase at a higher rate than public expenditure as response to the increase in income.

## Methods

In this current study, for addressing the objective, we intended to estimate to what extent private and public expenditures^c^ on health change as a response to changes in income level. Income (GDP per capita) elasticity on private and public spending on health was therefore estimated using multiple regression analyses. Data and estimation techniques are explained below.

### Data and variable

The present study analyzed annual panel data from 9 SEAR member countries (Bangladesh, Bhutan, India, Indonesia, Maldives, Nepal, Sri Lanka, Thailand and Timor-Leste) during the period 1995 -2010. The data on determinants of health expenditure used in the empirical analysis were sourced from the World Development Indicators and the data on health expenditure was extracted from National Health Accounts of the World Health organization [2,21]. It needs to be noticed that Myanmar, though a SEAR country, was excluded from the analyses as data on some independent variables were missing.

In this study, total per capita health expenditure and such expenditure separated into per capita public and private health expenditure were predicted by national income (per capital GDP at international dollar and at constant price), demographic structure (share of female, share of elderly population aged 65 years and above as well as share of urban inhabitants in total population) and health condition (life expectancy at birth in years).

### The models and estimation strategy

Panel data (also known as longitudinal or cross sectional time-series data) is a dataset in which the behavior of entities (states, companies, individuals, countries, etc.) is observed across time. Panel data allows us to control for variables we cannot observe or measure like variables that change over time but not across countries [22]. In this study, using a strongly balanced panel data with 9 SEAR countries over 16 years, fixed-effect model (FEM) and random effect models (REM) were employed for estimation.

The estimations were made using three dependent variables separately, i.e. total, public and private health expenditure per capita. The explanatory variables included national income level, demographic structure and health condition. Such variables were used in earlier studies for predicting health expenditure or related ones [5,17,19,20,23]. Decision on health expenditure is normally followed by income change. We, therefore, applied even dynamic models including three-year lagged data on national income while estimated health expenditure per capita [17,20,23].

### Fixed effect model (FEM)

Fixed-effect model represents the observed quantities in terms of explanatory variables that were treated as if the quantities were non-random. Equation (1) expresses the model considering subscripts *i* and *t* were used for indicating country and year respectively in a panel data set:

1 $$ {y}_{it}=\beta {x}_{it}+{\alpha}_i+{u}_{it} $$

where ${y}_{it}$ was the dependent variable (natural log of health expenditure per capita), *x*_*it*_ was natural log of GDP per capita and a number of control variables, namely, demographic structure (female population, urban population and elderly population as a percentage of total population) health condition (life expectancy at birth). $\beta$ was the coefficient for any independent variable, α_i_ represented unknown intercept for any entity (country) and ${u}_{it}$ was the error term.

### Random-Effect Model (REM)

The random effect model, unlike the fixed effect model, assumed the variation across countries to be random and uncorrelated with the predictor or independent variables included in the model. If we had reason to believe that differences across entities (countries) had some influence on our dependent variable then we should have used random effect. An advantage of random effect model was that we could include time invariant variables. The random effect model is as follows:

2 $$ {y}_{it}=\beta {x}_{it}+{a}_i+{u}_{it}+{\in}_{it} $$

where, $u$ was a within country error and ε is a between country error, remaining notations were same as the fixed effect model expressed in equation (1). Random effect assumed that the countries error term was not correlated with the predictors which allowed for time-invariant variables to play a role as explanatory variables. Random effect model was estimated by Generalized Least Squares (GLS) while fixed effect model was estimated by ordinary least squares with differential intercept dummies for country and year dimensions. The Hausman specification test was carried out to choose between fixed-effect and random-effect models.

## Results

### Descriptive statistics

**Figure 1:**
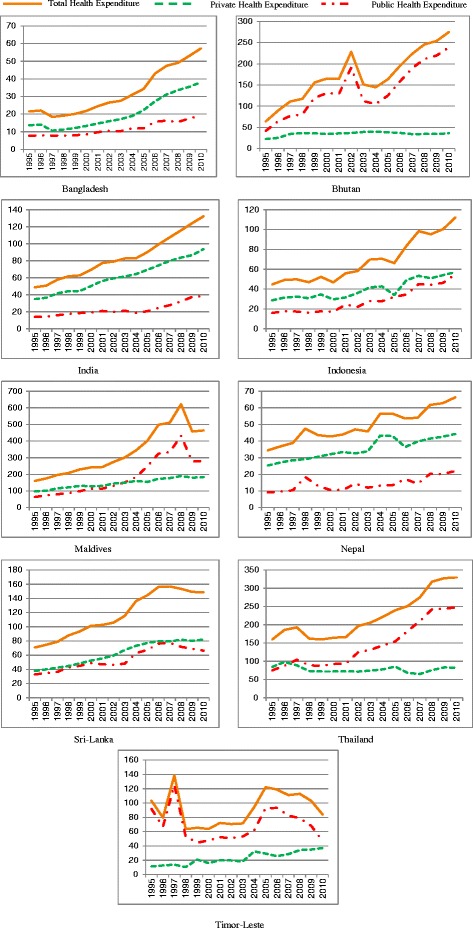
**Trend of health expenditure per capita (PPP adjusted US$) in SEAR Countries over 1995 -2010.**

Table 1 shows the descriptive statistics of the variable of interest in SEAR countries. Average values of the variables in 1995-2010 with 95% confident interval, and number of observations (on the basis of which the statistics were calculated) were presented for each country separately as well as for all countries. The income of the countries ranged between US$ 938.4 (Nepal) and US$ 6,355.6 (Thailand). Among these 9 countries Maldives experienced the largest total health expenditure per capita (US$ 333.0) while Bangladesh had the lowest expenditure (US$ 32.3). The trends of public and private expenditure as well as their total in each country were presented in Figure 1. Demographic characteristics showed that some countries had higher proportion of female (Nepal, Sri Lanka and Thailand) while most of the countries had higher number of male population. Indonesia experienced the largest urbanization with 43.9% population in the urban areas. Nepal on the contrary had only 14.3% urban people. Thailand had largest proportion of elderly population (7.5%), followed by Sri Lanka (7.0%). Lowest proportion of elderly was observed in Timor-Leste (2.6%). Life expectancy at birth was high in Thailand and Sri Lanka with 73.0 years and 72.3 years respectively, when Timor-Leste had the lowest (57.8 years).

**Table 1:** **Variables employed in the analysis by countries**

**Variables** ^**1)**^	**Country**									
**Health expenditure per capita** ^**2)**^	**Bangladesh**	**Bhutan**	**India**	**Indonesia**	**Maldives**	**Nepal**	**Sri Lanka**	**Thailand**	**Timor-Leste**	**All**
*Public*	12.5	137.6	29.0	39.5	226.3	21.0	64.5	166.1	84.6	86.8
	(10.2-14.7)	(105.8-169.4)	(23.3-34.8)	(31.5-47.4)	(161.2-291.3)	(16.6-25.3)	(54.6-74.3)	(129.8-202.4)	(72.6-96.5)	(72.5-101.0)
*Private*	19.9	34.1	54.8	29.3	106.8	28.6	52.8	56.1	7.6	43.3
	(15.0-24.7)	(31.7-36.6)	(46.7-63.0)	(25.1-33.5)	(95.8-117.7)	(27.4-29.7)	(45.7-59.9)	(50.5-61.7)	(6.2-9.1)	(38.5 -48.2)
*Total*	32.3	171.7	83.89	68.8	333.0	49.5	117.2	222.2	92.2	130.1
	(25.3-39.39)	(139.2-204.3)	(70.1-97.7)	(56.8-80.7)	(257.7-408.3)	(44.4-54.6)	(100.4-134.0)	(189.5-255.0)	(79.4-105.0)	(112.4-147.8)
GDP per capita^2)^	1123.1	3348.2	2106.8	3090.3	5374.2	938.4	3435.6	6355.6	1077.5	3034.5
	(1003.4-1242.8)	(2871.7-3824.7)	(1813.4-2400.3)	(2842.4-3338.1)	(4591.1-6157.2)	(893.8-982.9)	(3049.9-3821.3)	(5919.0-6792.2)	(986.7-1168.3)	(2717.4-3351.6)
Female population, %^3)^	48.9	48.2	48.2	50.1	49.6	50.3	50.3	50.7	48.9	49.4
	(48.8-49.0)	(47.7-48.7)	(48.2-48.3)	(50.0-50.1)	(49.2-49.4)	(50.2-50.3)	(50.2 - 50.5)	(50.6-50.8)	(48.8-48.9)	(49.3 -49.6)
Urban population, %^3)^	24.9	28.4	28.7	43.9	32.1	14.3	15.5	31.9	25.4	27.2
	(23.8-26.0)	(25.9-30.9)	(27.9-29.5)	(41.6-46.3)	(29.4-34.8)	(13.3-15.2)	(15.3-15.8)	(31.3-32.6)	(24.4-26.3)	(25.8 -28.7)
Elderly population, %^3)^	4.2	4.4	4.5	4.9	4.3	3.7	7.0	7.5	2.6	4.8
	(4.1-4.3)	(4.2-4.5)	(4.3-4.6)	(4.7-5.2)	(3.9-4.7)	(3.6-3.9)	(6.7-7.4)	(7.0-8.1)	(2.4-2.7)	(4.5-5.1)
Life expectancy at birth, years	65.9	62.9	62.7	66.6	72.2	63.7	72.3	73.0	57.8	66.3
	(64.8-67.0)	(61.2-64.7)	(61.8-63.6)	(65.7-67.4)	(70.2-74.1)	(61.8-65.6)	(71.1-73.4)	(72.7-73.3)	(56.0-59.6)	(65.4-67.2)
N	16	16	16	16	16	16	16	16	(13-17)	144-153

Notes: ^1)^Mean and 95% confidence intervals are reported, ^2)^In international dollar, ^3)^As a percentage of total population.

### Correlation matrix

The correlation matrix of independent variables showed a rather simplistic description of the nature of the interrelationship among the independent variables, reported as being multicollinear problem (Table 2). None of the variables showed strong correlation with any other variables while referring to more than 0.85 as a high degree of relationship (correlation coefficient, r > 0.85) between variables for multicollinearity [24].

**Table 2:** **Correlation matrix of independent variables used in the analyses**

**Variables**	**GDP per capita**	**Female population**	**Elderly population**	**Urban population**	**Life expectancy at birth**
GDP per capita	1.000				
Female population	0.298 (0.0002)***	1.000			
Elderly population	0.708 (0.000)***	0.559 (0.000)***	1.000		
Urban population	0.457 (0.000)***	-0.109 (0.178)	0.113 (0.163)	1.000	
Life expectancy at birth	0.778 (0.000)***	0.491 (0.000)***	0.791 (0.000)***	0.221 (0.0062)***	1.000

Notes: ***, ** and * denotes significant at 1%, 5% and 10% risk level respectively.

### Econometric estimation

Estimated relationship between income and health expenditure in total and separated into public and private were presented in Tables 3 and 4. Hausman tests for identifying the better estimation model between FEM and REM showed that FEM was favored for estimating total and public expenditure, while any of FEM and REM could be appropriate for estimating private expenditure model [22]. GDP per capita showed positive effect on any of total, public and private expenditure on health. Regression coefficients suggested that the income-elasticity of total expenditure, i.e. one percent point increase in GDP per capita increased total and public expenditure by 0.73% and 0.412% respectively. The private expenditure on health increased by 1.128% in response to a one percent point increase in GDP per capita.

**Table 3:** **Estimated effect of national income** (**GDP per capita**) **on health expenditure per capita** ^**1**)^ **in SEAR countries**

**Determinants**	**Model 1**		**Model 2**		**Model 3**	
	**Total expenditure**		**Public expenditure**		**Private expenditure**	
	**FEM**	**REM**	**FEM**	**REM**	**FEM**	**REM**
Constant	- 9.442(0.000)***	- 8.312(0.000) ***	- 7.838(0.001)***	- 7.837(0.000)***	- 22.029(0.000)***	- 21.34(0.000)***
GDP per capita^1)^	0.7293(0.000) ***	0.7255(0.000)***	0.412(0.004)***	0.449(0.001)***	1.128(0.000)***	1.101(0.000)***
Female population	0.1087(0.002)**	0.0864(0.015)***	0.068(0.091)*	0.067(0.100)*	0.323(0.000)***	0.309(0.000)***
Urban population	0.0240(0.001)***	0.0205(0.002)**	0.026(0.002)***	0.027(0.000)***	0.014(0.101)*	0.010(0.208)
Elderly population	0.0749(0.041)*	0.0634(0.089)*	0.206(0.000)***	0.192(0.000)***	- 0.301(0.000)***	- 0.288(0.000)***
Life expectancy at birth	0.0297(0.001)***	0.0323(0.000)***	0.054(0.000)***	0.052(0.000)***	0.029(0.009)***	0.032(0.002)***
Observation (N)	140	140	140	140	140	140
R^2^(R-squared)						
*Within*	0.8651	0.8643	0.8752	0.8750	0.6859	0.6853
*Between*	0.4924	0.5093	0.2837	0.2974	0.4195	0.4462
*Overall*	0.5501	0.5666	0.3875	0.4005	0.4346	0.4590
F-statistics	161.61(0.000)***	-	176.66 (0.000)***	-	55.04(0.000)***	-
Wald chi^2^ (5)	-	686.58 (0.000)***	-	830.37(0.000)***	-	280.63 (0.000)***
Hausman test	Chi^2^ (5) = 26.37 (0.0001)***		Chi^2^ (5) = 13.11 (0.022)*		Chi^2^ (5) = 4.04 (0.5431)	

Notes: ^1)^ Natural logged, ***, ** and * denotes significant at 1%, 5% and 10% risk level respectively.

**Table 4:** **Estimated effect of national income** (**natural logged GDP per capita**) **considering three**-**year lagged sustainable income change as well as demographic structure and health condition on health expenditure per capita** ^**1**)^ **in SEAR countries**

**Explanatory variables**	**Model 1**		**Model 2**		**Model 3**	
	**Total expenditure**		**Public expenditure**		**Private expenditure**	
	**FEM**	**REM**	**FEM**	**REM**	**FEM**	**REM**
Constant	- 10.579(0.000)***	- 4.698(0.028)***	- 9.009(0.000)***	- 11.218(0.001)***	-22.458(0.000)***	- 2.669(0.310)
GDP per capita^1)^	0.918(0.002)***	1.862(0.018)***	0.684(0.058)**	2.001(0.114)	1.452(0.000)***	1.939(0.046)**
Lag 1 of GDP per capita^1)^	- 0.062(0.834)	- 0.429(0.598)	- 0.155(0.668)	0.007(0.996)	- 0.239(0.495)	- 1.221(0.224)
Lag 2 of GDP per capita^1)^	- 0.034(0.734)	- 0.026(0.927)	- 0.012(0.924)	- 0.007(0.988)	- 0.057(0.625)	- 0.059(0.868)
Lag 3 of GDP per capita^1)^	0.036(0.640)	0.009(0.967)	0.022(0.816)	- 0.158(0.634)	- 0.016(0.858)	0.217(0.395)
Female population	0.128(0.000)***	- 0.005(0.915)	0.092(0.035)**	0.083(0.234)	0.326(0.000)*	- 0.057(0.291)
Urban population	0.025(0.002)***	- 0.025(0.000)***	0.030(0.002)***	- 0.026(0.000)***	0.008(0.402)	- 0.025(0.000)***
Elderly population	0.061(0.135)	- 0.234(0.000)***	0.197(0.000)***	- 0.316(0.000)***	- 0.319(0.000)***	- 0.151(0.001)***
Life expectancy at birth	0.018(0.139)	0.005(0.680)	0.038(0.012)***	- 0.015(0.421)	0.036(0.014)***	0.054(0.000)***
Observation (N)	125	125	125	125	125	125
R^2^(R-squared)	-	-	-	-	-	-
*Within*	0.8548	0.3816	0.8542	0.2026	0.6807	0.3627
*Between*	0.4869	0.8485	0.2897	0.7167	0.4507	0.7119
*Overall*	0.5363	0.8037	0.3936	0.7091	0.4500	0.6629
F-statistics	79.46(0.000)***	-	79.08 (0.000)***	-	28.78(0.000)***	-
Wald chi^2^ (5)	-	475.01(0.000)***	-	282.81(0.000)***	-	228.11(0.000)***
Hausman test	Chi^2^ (8) =102.88 (0.000)***		Chi^2^ (8) =108.51 (0.000)***		Chi^2^ (8) =104.02 (0.000)***	

NB: ^1)^ Natural logged, ***, ** and * denotes significant at 1%, 5% and 10% risk level respectively.

The control variables i.e. demographic structure (share of female, elderly population and urban population) and health condition (life expectancy at birth) showed expected effect on any kind of health expenditures. It was observed that share of elderly population in total population had positive effect on total and public expenditure, but negative effect on private expenditure on health. When included three-year lagged variable of income level (Table 4), we still found in the fixed effect model that the public spending in the reporting years was income-inelastic (0.918) and the private spending even more elastic (1.452).

## Discussion

Findings from the current analysis showed that the public expenditures were inelastic in relation to national income in SEAR countries, which implied that healthcare was a necessity for citizens in SEAR countries, while provided through the public sector. The private expenditure, on the contrary, was elastic, i.e. healthcare was considered as a luxury by the citizens when they could make their own decision mostly as individuals and households. Our models with three-year lagged data on national income did not show any significant change in income-elasticities on total and public health expenditure.

For estimating the income-elasticity of healthcare we constructed the regression models, based on a number of previously used models, where health expenditure had been predicted by national income while controlled for variations in demographic structure of the country and health condition [5-17,20,23]. This current study utilized published data on country level. These data were not sufficiently and appropriately available on some of the key control variables of interests. For instance, classification of age groups were based on ability to work (0-15, 16-64 and 64+ years), not focusing on health outcomes. Further, some previous studies controlled for literacy or education levels [25,26]. But we could not include this variable in this analysis as it was not found for all countries and years.

A good number of studies estimated income-elasticity of healthcare, but mostly using data from developed countries either employing cross-sectional or panel data [5-17]. These studies found a positive relationship between income and healthcare expenditure, but while some studies found healthcare a ‘necessity’, others found that a ’luxury’. Remarkably, very few studies utilized data from developing countries [16,20]. Further, studies that analyzed data from private and public sectors separately were rarely found [20]. Keeping the debate that if healthcare is a luxury or necessity in mind, Getnez (2000) investigated the relationship between income and healthcare expenditure using multi-level analysis and found that healthcare was a luxury at country-level and necessity at individual level [19].

The findings from the current study were comparable with some other studies [16,20]. Like Fager et. al. (2012) and Xu and Saksena (2011), we found that healthcare was a ‘necessity’ in low- and middle-income countries while total expenditure was considered in the estimation [16,20]. Xu and Saksena (2011) separated total health expenditure into public and private (precisely, out-of-pocket) like the current study [20]. The authors found that low-income countries considered healthcare as a ‘luxury’ and middle-income countries as a ‘necessity’ [20]. This current study found that SEAR countries, which ranged from low- to middle-income levels, regarded healthcare as a ‘necessity’ while delivered through public sector. In a static model, Xu and Saksena (2011) observed that income-elasticity of private healthcare (out-of-pocket expenditure) was more than unity (1.098) in low-income countries and closed to unity (0.842-0.869) in the middle-income countries [20]. However, income-elasticities reduced significantly in the dynamic model in low-income and middle-income countries. Our current study consistently found that healthcare through private sector was income-elastic, in both static and dynamic models (Tables 3 and 4), implying that healthcare was a ‘luxury’.

National Health Accounts showed that public expenditure generally contributed less to the total health expenditure in a number of SEAR countries [2]. The private expenditure, mostly spent as out-of-pocket payments, was the most common mechanism for healthcare funding in these countries. Introducing the healthcare financing mechanisms, which can reduce the burden of out-of-pocket payments to an affordable level for citizens, appeared to be a challenge in many low- and middle-income countries, including those in SEAR though it is a fundamental component of universal health coverage. Political commitment is considered to be essential in this regard. It can be argued that increasing public provisions for healthcare can increase the scope of redistribution of healthcare resources since such provision is financed by pooled fund, mostly taxes, where people from different income groups contribute. Redistribution from a pooled fund can be across rich and poor as well as across healthy and sick people [27]. The magnitude of redistribution depends on the distribution of healthcare utilization across different groups of people. This is often demonstrated that healthcare seeking behavior varies across socioeconomic groups and it is mostly pro-rich, however private sector is more pro-rich than the public sector [28,29]. Difference in healthcare seeking behavior across socioeconomic groups can be explained by variation in the degree of health awareness, physical access to healthcare facilities, economic hardship and so forth [29]. On the contrary, private provisions which are mostly occupied with out-of-pocket expenditure on healthcare lack redistributive capacity. It implies that a larger public sector with larger pooled fund will increase the scope of redistribution of healthcare resources across rich and poor as well as across healthy and sick people.

The Director General of the World Health Organization Dr. Margaret Chen in her speech in the Ministerial meeting on universal health coverage in February 2013 emphasized strongly on political commitment as well as an integrated effort at national level, saying that “Progress towards universal coverage cannot be achieved by health ministers acting alone, even in the presence of political commitment at the highest level of government. It requires a concerted national effort, with an especially close engagement of ministers of health and finance” [30].

Since previous studies as well as the current one indicated that citizens were more sensitive to private healthcare expenditure in relation with their income (income-elastic), governments in SEAR countries should seriously revisit the current way of involvement of private healthcare in healthcare financing system and try to reorganize both private and public sectors in a way so that equity in healthcare utilization across income-groups could be addressed. Such an action could address the political commitment and national effort towards universal health coverage.

## Conclusions

The citizens of SEAR countries consider healthcare as a necessity while provided through public sector and a luxury while delivered by private sector.

### Endnotes

^a^Structural Adjustment Programmes (SAPs) are economic policies for developing countries that have been promoted by the World Bank and International Monetary Fund (IMF) since the early 1980s by the provision of loans conditional on the adoption of such policies. Structural adjustment loans are loans made by the World Bank (source: http://www.who.int/trade/glossary/story084/en/, accessed on the 20th November 2014.

^b^An income elasticity greater than one usually means that a good is perceived as a “luxury” good and income elastic. An income elasticity between 0 and 1 is a normal good and income inelastic meaning that it does not respond as much to income changes and therefore is perceived as more of a necessity.

^c^Public health expenditure consists of recurrent and capital spending from government (central and local) budgets, external borrowings and grants (including donations from international agencies and nongovernmental organizations), and social (or compulsory) health insurance funds. Private health expenditure includes direct household (out-of-pocket) spending, private insurance, charitable donations, and direct service payments by private corporations (Source: World Development Indicators, The World Bank, 2014).
